# Accumulated subcutaneous fat in abdomen is associated with long COVID-19 symptoms among non-hospitalized patients: a prospective observational study

**DOI:** 10.3389/fmed.2024.1410559

**Published:** 2024-10-14

**Authors:** Tingxin Li, Baoming He, Yuping Liu, Chen Wang

**Affiliations:** ^1^Department of Health Management Center and Institute of Health Management, Sichuan Provincial People’s Hospital, School of Medicine, University of Electronic Science and Technology of China, Chengdu, China; ^2^Department of Neurology, Sichuan Provincial People’s Hospital, School of Medicine, University of Electronic Science and Technology of China, Chengdu, China; ^3^Ultrasound in Cardiac Electrophysiology and Biomechanics Key Laboratory of Sichuan Province, Sichuan Clinical Research Center for Cardiovascular Disease, Sichuan Provincial People’s Hospital, University of Electronic Science and Technology of China, Chengdu, China; ^4^Department of Cardiovascular Ultrasound and Noninvasive Cardiology, Sichuan Provincial People’s Hospital, University of Electronic Science and Technology of China, Chengdu, China

**Keywords:** long COVID, SARS-CoV-2, visceral fat, subcutaneous fat, non-hospitalized patients

## Abstract

**Introduction:**

Long COVID-19 symptoms may have a variety of potential overlapping causes. In this study, we aimed to investigate the potential correlation between abdominal adipose tissue and long COVID-19 symptoms in non-hospitalized patients in China.

**Methods:**

This is a prospective observational study. 424 subjects, recovered from COVID-19 for 2–4 weeks, were enrolled and 408 subjects were finished the follow-up investigation at baseline, 8^th^ week and 12^th^ week. Physical measurements were collected. Kaplan-Meier analysis and cox regression analysis were carried out to assess the correlation.

**Results:**

A total of 72 subjects reported the long COVID-19 symptoms. The adjusted Kaplan-Meier analysis and Cox regression analysis revealed a significant correlation with accumulated subcutaneous fat (SFA ≥ 2.0 dm^2^) and the long COVID-19 symptoms (*HR* = 2.63, *P* < 0.001 for male, *HR* = 1.52, P = 0.048 for female). However, overweight and central obesity showed positive correlation only in women.

**Discussion:**

This study suggested that accumulated subcutaneous fat in abdomen (SFA ≥ 2.0 dm^2^) was an important positive factor associated with long COVID-19 symptoms among Chinese non-hospitalized patients. Large investigation and prospective studies are needed to validate the correlation in the future.

## 1 Introduction

The global coronavirus disease 2019 (COVID-19) pandemic remains a great challenge for healthcare systems, despite the fact that the case fatality rate is decreasing (1). After early December 2022, the number of people infected with novel coronavirus in China increased sharply (2). Mental and physical health promotion and rehabilitation is more important than ever amid the continuing global fight against the pandemic (3).

The lasting symptom burden and impact of COVID-19 on patients has been examined in recent studies (4, 5). The long COVID-19 symptoms is defined by the UK‘s National Institute for Health and Care Excellence as “new or ongoing symptoms 4 weeks or more after the start of acute COVID-19”, which always be considered lasting more than 12 weeks (6). At least 65 million individuals around the world have long COVID-19 symptoms, based on a conservative estimated incidence of 10% of infected people and more than 651 million documented COVID-19 cases worldwide (7).

In COVID-19, obesity is considered to be a risk factor for adverse consequences. Depots of visceral fat (VF) are linked to the severity of COVID-19 in non-obese Asian people. The mechanisms underlying this relationship remain unknown (8). It has been noted that most of the analyses focused on hospitalized patients who were accurately diagnosed and obtained appropriate treatment and rehabilitation during the acute phase of illness (9). The vast majority of COVID-19 patients are treated at home (10). However, some researchers believe that most long COVID-19 symptoms cases are in non-hospitalized patients with mild acute illness (11). The incidence of long COVID-19 symptoms is 10–30% in non-hospitalized cases (12). The factors related to the long COVID-19 symptoms in mild illness patients were not well understood. Therefore, the aim of this study was to analyze the potential role of abdominal adipose tissue in the prolonged symptoms in Chinese non-hospitalized patients with SARS-CoV-2.

## 2 Materials and methods

### 2.1 Data source and sample selection

This preliminary study was conducted among adults who underwent physical health examinations at the Medical Examination Center of Sichuan Provincial People’s Hospital in China from January to April 2023, who recovered from SARS-CoV-2 for 2–4 weeks. The SARS-CoV-2 infection and recovery was confirmed by RT-PCR test or antigen test in each participant. We excluded patients who were hospitalized for COVID-19. Considering vaccines had an effect on reducing the risk of long symptom (13), all participants verbally confirmed that they had been vaccinated against COVID-19.

The study protocol was approved by the local Human Ethics and Research Ethics committees of Sichuan Provincial People’s Hospital in China (Approval No. 2020-97) and conducted in accordance with the human research ethical standards and regulations of the Helsinki Declaration (2014). All subjects provided written informed consent before participation in the study.

### 2.2 Data measurement and collection

Face-to-face comprehensive health interviews were conducted to assess the demographics, socioeconomic status, the details of infection, long COVID-19 complaints, and self-reported medical history, including hypertension, hyperglycemia, and so on. Standardized data forms were used to collect their self-reported long COVID-19 symptoms status at the 4^th^, 8^th^, and 12^th^ week of rehabilitation. Throughout the survey, trained health managers were responsible for the follow up by phone calls.

Physical measurements, including height, weight and waist circumference (WC), were recorded by trained medical staff. The BMI was calculated as weight in kilograms divided by height in meters squared (kg/m^2^). DUALSCAN HDS-2000, an abdominal dual bioelectrical impedance analysis (BIA) machine, was used to measure visceral fat area (VFA) and subcutaneous fat area (SFA) around the abdomen (OMRON Healthcare Co., Kyoto, Japan). The test subjects were required to empty their bowels and bladders and remove jackets, watches, necklaces, and other metal objects. According to the Chinese Guidelines for Prevention and Control of Adult Overweight and Obesity, overweight was defined as BMI between 24 kg/m^2^ and 28 kg/m^2^. And obesity was defined as BMI ≥ 28 kg/m^2^. Central obesity in Chinese was defined as WC ≥ 85 cm in female or WC ≥ 90 cm in male. Accumulated subcutaneous fat in abdomen was defined as SFA ≥ 2.0 dm^2^ and accumulated visceral fat was defined as VFA ≥ 1.0 dm^2^.

Fasting blood samples were collected from participants after overnight fasting for at least 8 h. All blood samples were sent to the laboratory of Sichuan Provincial People’s Hospital to test the biochemical indicators, fasting blood glucose (FBG), lymphocyte number (LYM), total protein (TP), triglycerides (TG), total cholesterol (TC), HDL-cholesterol (HDL-C), and LDL-cholesterol (LDL-C).

### 2.3 Statistical analyses

The long COVID-19 symptoms in Chinese non-hospitalized patients with SARS-CoV-2 and the potential relevant factors were unclear. Statistical sample size calculation by PASS software (version 20.0.6). The enrollment duration of 3 months, follow-up period of 3 months, two-tailed α = 0.05, power = 0.9, primary endpoint was ongoing long COVID-19. It was reported the incidence of long COVID-19 was 10–30% in non-hospitalized cases (12). We predict 10% as a conservative estimate incidence in this group. Refer to the latest research results, the incidence was 13.25% in unexposed group (with normal adipose tissue) and 43.69% in the exposure group (with excessive adipose tissue) respectively (14). The minimum sample size was 388 calculated according to the method of Cox Proportional Hazards Regression. Lost participants would be treated as censored data. For inference statistics, the linear model ANOVA was used for continuous data and the chi-square test was used for categorical data. Kaplan-Meier analysis and Cox regression analysis were used to identify independent risk factor for the primary outcome and determine the hazard ratios (HRs) and 95% confidence intervals (CIs) in different genders. All *P*-values were two-sided, and *P* < 0.05 indicated statistical significance. All statistical analysis were carried out using the Statistical Package for Social Science (SPSS) software (version 17.0, IBM, Armonk, NY, USA).

## 3 Results

### 3.1 Characteristics of the study population

**FIGURE 1:**
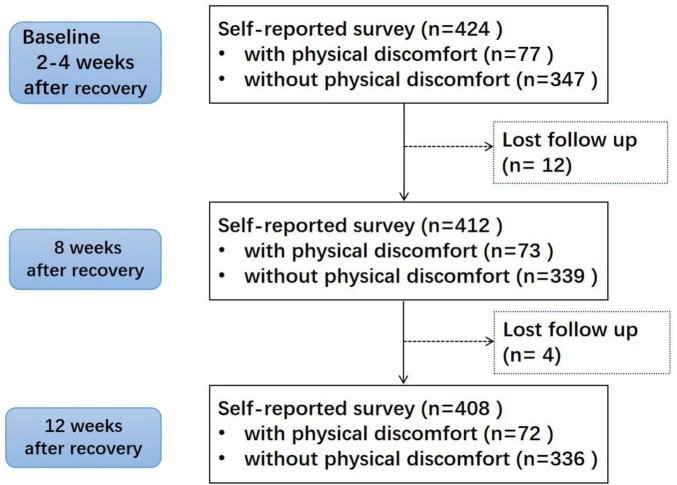
Study flowchart.

A total of 424 participants were enrolled, including 221 males and 203 females. 16 patients did not finish the survey. It was assumed that their reason for withdrawal was unrelated to their risk of experiencing the event of interest. They were treated as censored data in Kaplan-Meier analysis and Cox regression analysis. Figure 1 shows the flow chart of the research. The mean age was 41.56 ± 10.70 years, and BMI averaged 25.57 ± 4.12 kg/m^2^. The rate of obesity, overweight and central obesity in males were higher than females. Characteristics of the study subjects were shown in Table 1. The characteristics in different genders were shown in Supplementary Table 1.

**TABLE 1:** Clinical characteristics of participants.

Characteristics	Number (*n* = 424)
Age (years), mean (SD)	41.56 (10.70)
Male (%)	221 (52.1)
Married (%)	365 (86.1)
Obesity (%)	82(19.3)
Overweight (%)	177 (41.7)
Central obesity (%)	171 (41.9)
SFA ≥ 2.0 dm^2^ (%)	194 (45.8)
VFA ≥ 1.0 dm^2^ (%)	207 (48.8)
**Nationality (%)**	
Han	274 (64.6)
Tibetan	142 (33.5)
Other	8 (1.9)
**Smoking status (%)**	
Never smoker	336 (79.2)
Ex-smoker	14 (3.3)
Current smoker	74 (17.5)
**Drinking status (%)**	
Never	259 (61.1)
Occasionally	135 (31.8)
Regularly	30 (7.1)
**History of disease (%)**	
Hypertension	27 (6.4)
Diabetes	10 (2.4)
Surgical history	59 (13.9)
None	328 (77.4)
BMI (kg/m^2)^, mean (SD)	25.57 (4.12)
WC (cm), mean (SD)	86.46 (11.00)
VFA (dm^2^), mean (SD)	1.06 (0.46)
SFA (dm^2^), mean (SD)	1.97 (0.69)
SBP (mmHg), mean (SD)	124.25 (46.84)
DBP (mmHg), mean (SD)	73.28 (11.13)
Total protein (g/L), mean (SD)	73.46 (3.94)
LYM (10^9^/L), mean (SD)	2.08 (0.59)
TG (mmol/L), mean (SD)	1.68 (1.60)
TC (mmol/L), mean (SD)	4.76 (0.97)
HDL-C (mmol/L), mean (SD)	1.26 (0.31)
LDL-C (mmol/L), mean (SD)	2.94 (0.85)
FPG (mmol/L), mean (SD)	4.85 (0.64)

VFA, visceral fat area; SFA, subcutaneous fat area; SBP, systolic blood pressure; DBP, diastolic blood pressure; LYM, number of lymphocyte; FPG, fasting plasma glucose.

### 3.2 Correlation analysis between indicators and ongoing long COVID-19 symptoms with valid data

The discomforts which continued for some time after the rehabilitation of COVID-19 were called ongoing symptoms, including the cardiovascular, respiratory, neurological, musculoskeletal, metabolic, as well as psychiatric problems, generalized pain, fatigue etc. The follow-up symptom cases among the participants were shown in the Supplementary Table 2. In this manuscript, the long COVID-19 symptoms was defined as new or ongoing symptoms 12 weeks or more from the recovery of acute COVID-19. At the end of 12 weeks of rehabilitation, 72 subjects were still self-reported with one or more long COVID-19 symptoms. The associations between physical examination indicators and ongoing symptom among 408 valid data were presented in Table 2. 34.7% (25/72) long COVID-19 patients had history disease, such as hypertension, diabetes, surgical history and so on. The incidence in Han people (21.7%, 56/258) was high than Tibetans (9.9%, 14/142). Single-factor analysis showed higher SFA of abdominal and overweight were associated with long COVID-19. No correlation was observed in other clinical indicators.

**TABLE 2:** Indicators of the population with long COVID-19 symptoms.

Characteristics	Total (*n* = 408)	With symptoms (*n* = 72)	Without symptoms (*n* = 336)	*P*-value
Male (%)	214 (52.5)	34 (15.9)	180 (84.1)	0.36^a^
With disease history (%)	96 (23.5)	25 (26.0)	71 (74.0)	0.02^a^
Han (%)	258 (63.2)	56 (21.7)	202 (78.3)	0.01^a^
Tibetan (%)	142 (34.8)	14 (9.9)	128 (90.1)	
Current smoker (%)	73 (17.9)	12 (16.4)	61 (83.6)	0.95^a^
Regularly drinking (%)	30 (7.4)	8 (26.7)	22 (73.3)	0.20^a^
Age (years), mean (SD)	41.63 (10.82)	40.92 (12.41)	41.78 (10.47)	0.54^b^
BMI (kg/m^2)^, mean (SD)	25.59 (4.18)	25.45 (4.35)	25.62 (4.15)	0.76^b^
Obesity (%)	81(19.9)	11 (13.6)	70 (86.4)	0.04^a^
Overweight (%)	167 (40.9)	39 (23.4)	128 (76.6)	
WC (cm), mean (SD)	87.28 (11.62)	86.50 (10.38)	86.55 (11.30)	0.97^b^
Central obesity (%)	171 (41.9)	27 (15.8)	144 (84.2)	0.43^a^
VFA (dm^2^), mean (SD)	1.08 (0.50)	1.00 (0.37)	1.12 (1.09)	0.38^b^
VFA ≥ 1.0 dm^2^ (%)	195 (47.8)	35 (17.9)	160 (82.1)	0.89^a^
SFA (dm^2^), mean (SD)	1.96 (0.69)	2.32 (0.80)	1.89 (0.64)	<0.001^b^
SFA ≥ 2.0 dm^2^ (%)	182 (44.6)	46 (25.3)	136 (74.7)	<0.001^a^
SBP (mmHg), mean (SD)	124.40 (47.70)	121.43 (15.84)	125.04 (52.04)	0.56^b^
DBP (mmHg), mean (SD)	73.31 (11.25)	74.22 (11.18)	73.12 (11.27)	0.45^b^
FPG (mmol/L), mean (SD)	4.86 (0.64)	4.89 (0.77)	4.86 (0.61)	0.66^b^
TG (mmol/L), mean (SD)	1.66 (1.59)	1.50 (0.91)	1.69 (1.69)	0.35^b^
TC (mmol/L), mean (SD)	4.76 (0.97)	4.74 (0.90)	4.77 (0.98)	0.82^b^
HDL-C (mmol/L), mean (SD)	1.26 (0.31)	1.29 (0.29)	1.26 (0.31)	0.40^b^
LDL-C (mmol/L), mean (SD)	2.95 (0.85)	2.95 (0.80)	2.95 (0.87)	0.99^b^
Total protein (g/L), mean (SD)	73.57 (3.68)	73.60 (4.31)	73.52 (3.53)	0.88^b^
LYM (10^9^/L), mean (SD)	2.05 (0.56)	2.11 (0.57)	2.07 (0.56)	0.59^b^

VFA, visceral fat area; SFA, subcutaneous fat area; SBP, systolic blood pressure; DBP, diastolic blood pressure; LYM, number of lymphocyte; FPG, fasting plasma glucose.

^a^*P*-value of chi-square test,

^b^*P*-value of ANOVA test.

For the differences of baseline (see Supplementary Table 1), subgroup analysis in different genders was done (see Supplementary Table 3). The related factors of long COVID-19 in different genders showed differences. Disease history, nationality, overweight, central obesity, SFA were showed association with the incidence in males. Only central obesity and SFA showed statistical significance in females.

Previous studies had shown that there are great genetic differences between Tibetans and Hans. Subgroup analysis in different nationality was done (see Supplementary Table 4). Overweight, obesity, SFA were showed association with the incidence in both Hans and Tibetans. Disease history showed statistical differences only in Han, while age and gender showed statistical differences only in Tibetans.

### 3.3 Kaplan-Meier analysis and cox regression analysis

**FIGURE 2:**
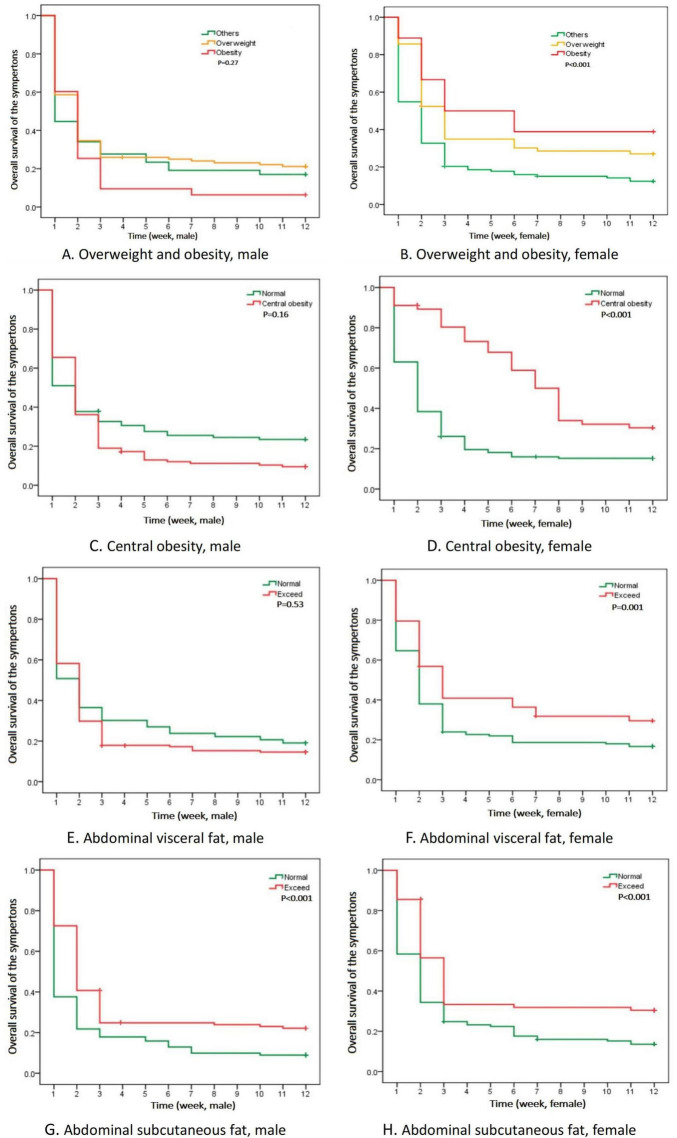
Kaplan-Meier analysis of time curve with long COVID-19 symptoms in different genders. **(A,B)** Time of the symptoms of overweight and obesity; **(C,D)** Time of the symptoms of central obesity; **(E,F)** Time of the symptoms of abdominal visceral fat; **(G,H)** Time of the symptoms of abdominal subcutaneous fat.

There was a great difference in the time for adverse reactions to subside in patients of different genders. The time curve of long COVID-19 symptoms was drawn by Kaplan-Meier analysis (Figure 2). Considering the results of statistical analysis and known clinical expertise as a whole, the variables including age, nationality and disease history to be included in the Cox regression equation were determined. Multivariate Cox regression analysis was carried out to assess the association between long COVID-19 symptoms and those factors in different genders: overweight, obesity, central obesity, VFA ≥ 1.0 dm^2^ and SFA ≥ 2.0 dm^2^. In the adjusted model, only accumulated subcutaneous fat (SFA ≥ 2.0 dm^2^) showed positive correlation with long COVID-19 symptoms in both males (*HR* = 2.63, *P* < 0.001) and females (*HR* = 1.52, *P* = 0.048). While, central obesity and overweight showed positive correlation only in females. Except the accumulated subcutaneous fat, no significant difference was found among men. It seemed that accumulated subcutaneous fat (SFA ≥ 2.0 dm^2^) was an statistically significant factor associated with long COVID-19 symptoms in those non-hospitalized patients (Table 3). The sensitivity analysis supports these findings.

**TABLE 3:** Multivariate Cox regression analysis of the long COVID-19 symptoms^#^.

Gender	Factors (n/cases)	*P-*value	*HR*	*95.0% CI*
Male	Overweight (22/34)	0.22	0.68	0.36–1.27
	Obesity (4/34)	0.06	0.71	0.48–0.90
	Central obesity (11/34)	0.058	0.88	0.66–1.21
	VFA ≥ 1.0 dm^2^ (22/34)	0.73	1.08	0.68–1.72
	SFA ≥ 2.0 dm^2^ (25/34)	< 0.001	2.63	1.85–3.73
Female	Overweight (17/38)	0.08	2.03	0.92–4.49
	Obesity (7/38)	0.62	1.21	0.57–2.57
	Central obesity (17/38)	0.048	1.68	0.96–2.93
	VFA ≥ 1.0 dm^2^ (13/38)	0.76	1.02	0.58–1.80
	SFA ≥ 2.0 dm^2^ (21/38)	0.048	1.52	1.00–2.32

HR, hazard ratio; CI, confidence interval; VFA, visceral fat area; SFA, subcutaneous fat area. **^#^**Adjusted age, disease history and nation.

## 4 Discussion

In our observational study, 72 subjects among non-hospitalized patients, including 34 males, reported having one or more symptom at the end of 12-week follow-up. It was consistent with previously published reports among hospitalized patients (12). SFA showed a potential risk of long COVID-19 symptoms in mild illness patients. There were likely multiple, potentially overlapping causes of long COVID-19 symptoms. Several hypotheses for its relationship had been suggested, including the severity of the acute illness (15), nutritional level (16), obesity or physical performance (17), biopsychosocial effects (18), and so on. Mechanistic studies were generally at an early stage, and many questions remain to be addressed. At present, most studies come from hospitalized patients (15), and the post-infection symptoms of mild patients were still unclear. However, with the constant variation of virus and the increase of population immunity, at least 65 million individuals worldwide were estimated to have long COVID-19 symptoms, with cases increasing daily (19). To manage and control long COVID-19 symptoms was a challenge for the health system.

Among the methods of measuring VFA, waist circumference could be used for convenience. However, waist circumference includes both visceral and subcutaneous fat, and it could vary even at the same measures of waist circumference (20). In this prospective observational study, after conducting adjusted Cox regression analysis, we found a significant association between central obesity (waist circumference ≥ 85 cm in female or waist circumference ≥ 90 cm in male) and the long COVID-19 symptoms only in female patients, despite the initial significant correlation observed between syndrome prevalence and waist circumference in both males and females during the one-way ANOVA analysis. For further identification, an abdominal dual Bioelectrical impedance analysis (BIA) machine was used to measure VFA and SFA around the abdomen (OMRON Healthcare Co., Kyoto, Japan). This was a noninvasive, cost-effective, and widely accepted method for nutritional evaluation and anthropometric measurements. Of course, using computed tomography (CT), magnetic resonance imaging (MRI) or dual-energy X-ray absorptiometry (DXA), more direct methods had been developed to measure abdominal fat distribution. However, these methods are expensive or expose individuals to radiation, which could not be widely used in general health screening. BIA could be a good alternative tool to assess abdominal fat composition because of its simplicity and noninvasiveness. Several studies had established the validity of evaluating body fat composition with BIA versus CT scans or DEXA, and these studies had demonstrated a high degree of concordance between BIA and CT scans or DEXA (21, 22). However, BIA had some limitations, such as the results may be influenced by gender, race/ethnicity, drugs, and edematous condition, which need to be paid attention to in the future.

Adipose tissue was a highly active endocrine organ that produces and secretes a variety of metabolically and immunologically active molecules called adipokines. The importance of adipokines, such as leptin, resistin and adiponectin in regulating insulin sensitivity and metabolism, is well acknowledged (23). A prospective observational study reported that high concentrations of resistin were associated with worse clinical outcomes and more pronounced inflammation in COVID-19 patients, while leptin and adiponectin were not (24). In the past, visceral fat content was often expressed by waist circumference or visceral fat index. Previous researchers, such as Korac et al. (25), showed that the effects of SFA and VFA on resistin expression could be influenced by obesity (25). Significant differences in resistin levels were observed in obese women; resistin gene expression was higher in VAT and SAT of obese, compared to normal-weight women (25). In Chinese subjects, visceral fat mass was always high, but adipokines (adiponectin and resistin) were diversely associated with insulin resistance (26). The amount of visceral fat was considered in accord with serum resistin level (27). However, in clinical practice, it was found that waist circumference and visceral fat index showed their own limitations in evaluating visceral fat accumulation (28). Many novel surrogate indicators of adipose accumulation were concerned, such as waist toheight ratio (29), weight-adjusted waist index (30), metabolic score for visceral fat (31), and so on. With the update of detection methods, BIA, CT scans or DEXA were more directly to evaluate visceral fat. Recent researchers showed, regardless of normal-weight and obese women of COVID-19 patients, the expression levels of resistin were high in SF, but leptin and adiponectin are both low (24). Our study found accumulated subcutaneous fat in abdomen (SFA ≥ 2.0 dm^2^) was an important positive factor associated with long COVID-19 symptoms in both males and females. It might be related to the high level of resistin expression. It has been hypothesized that once subcutaneous adipose tissue reached its maximal expanding capacity, fatty acids redistributed ectopically in less insulin sensitive visceral adipose tissue and non-adipose tissues, causing lipotoxic effects and insulin resistance (32). Even if the pathophysiological significance of abdominal subcutaneous adipose tissue to metabolic risk has been confirmed in many correlation and epidemiologic studies (33, 34), the correlation of this abdominal adipose tissue storage dysfunction with long COVID-19 symptoms is still unclear. The difference in leptin, adiponectin, and resistin gene/protein expression in abdominal visceral or subcutaneous adipose tissue and their relationship with long COVID-19 symptoms risk merits be discussed. Endocrine dysfunction of abdominal SFA might be a risk sign of long COVID-19 symptoms.

Another thing worth exploring was angiotensin-converting enzyme (ACE) 2 and long COVID-19 symptoms. In patients with severe or morbid obesity, compared with SF, the VF showed a higher expression of angiotensin-converting enzyme ACE2 (35), a primary receptor of SARS-CoV-2 (36). ACE2 expression and/or polymorphism could influence both the susceptibility of people to SARS-CoV-2 infection and the outcome of COVID-19 disease (37, 38). But the relationship of ACE2, VF and long COVID-19 symptoms in mild obesity or overweight population was still vague. Use of ACE inhibitor or angiotensin receptor blockers for the purpose of treating hypertension in patients with COVID-19 did not lower the likelihood of severe COVID-19 (39). No association was observed between the ACE2 expression levels and anosmia in a cohort of COVID-19 hospitalized patients (40). It was rather unlikely that VF would serve as a reservoir for SARS-CoV-2 in non-severe COVID-19 (41). In this study, all patients were non-hospitalized and had a low weight base (BMI = 25.57 ± 4.12 kg/m^2^). The accumulated subcutaneous fat (SFA ≥ 2.0 dm^2^), but not visceral fat (VFA ≥ 1.0 dm^2^), showed a strong correlation with long COVID-19 symptoms. This is the first report to shift the focus from visceral fat to subcutaneous fat. Although it can’t be sure whether this is associated with racial genetic, mild COVID-19 without hospitalization, or the influence of specific virus strains, the potential function and activity of SF, such as expression of ACE2 and adipokines, in long COVID-19 symptoms patients need more attention. Further research is needed to clarify in the future.

The study had several limitations. This was a observational research in a specific range of population and the symptom description collected from self-reporting without further verification in clinical examination or laboratory testing; thus, strong causal inferences cannot be made. Second, the abdominal fat distribution data in this analysis was from BIA, which could be influenced by gender, race/ethnicity, drugs, and edematous conditions. Third, although the population included in this study was promised to be vaccinated with COVID-19 vaccine, they couldn’t accurately describe their vaccine types. Therefore, it was not known whether different vaccines had an impact on this research. Moreover, psychological and emotional interference was not ruled out in this analysis. And because of financial constraints, it is a pity that no data of ACE2, leptin, adiponectin and resistin gene/protein were collected. There were few patients at the end point (72 subjects), we did not carry out more sophisticated multivariable analysis further. These should be supplemented by future research.

Despite these limitations, there are several strengths in this manuscript. Previous studies suggested an association between obesity (especially visceral fat) and severe COVID-19. This prospective observational study focused on COVID-19 patients who are not hospitalized. 16.98% of non-hospitalized patients reported with long COVID-19 symptoms at 12^th^ week. And the results of this study firstly suggested a significant association between accumulated SF in abdomen and long COVID-19 symptoms. Shifting the focus of research on long COVID-19 from VF to SF may provide a new research idea for preventing long COVID-19 symptoms. In any case, further investigation is desirable.

## 5 Conclusion

A total of 16.98% of non-hospitalized patients reported long COVID-19 symptoms. Accumulated subcutaneous fat in abdomen (SFA ≥ 2.0 dm^2^) had positive correlation with symptoms in these participants. Central obesity and overweight showed potential risk only in females. No significant correlation was found in obesity or other physical indicators.

Our finding could have some implications for how healthcare systems prevent and support long COVID-19 symptoms patients. Although existing research is not enough to reduce morbidity and improve outcomes for people with long COVID-19 symptoms, it may provide a new research idea for prevention. More research would be built on the basis of this study. As a long-term goal, to respond to long COVID-19 symptoms crisis, we need robust policies and funding to train and educate the health-care and research workforce, and focus on the new research and care in this area.

## Data Availability

The raw data supporting the conclusions of this article will be made available by the authors, without undue reservation.
