# Supplementary material for: Accumulated subcutaneous fat in abdomen is associated with long COVID-19 symptoms among non-hospitalized patients: a prospective observational study
Source: Front Med (Lausanne). 2024 Oct 14;11:1410559. doi: 10.3389/fmed.2024.1410559 (PMC11514070; doi:10.3389/fmed.2024.1410559)
Supplement: Supplementary file 1 [file Data_Sheet_1.pdf]

Supplemented Table 1-1 Clinical characteristics in different genders

Characteristics	Male (n =221)	Female (n = 203)	*F/chi-square* value	*P* value
Age (years), mean (SD)	40.88(10.35)	42.30(11.05)	1.87	0.17[b]
Married (%)	182 (82.4)	183 (90.2)	5.36	0.024[a]
Obesity (%)	64 (29.0)	18 (8.9)		
Overweight (%)	109 (49.3)	68 (33.5)	63.51	<0.001[a]
Others (%)	48 (21.7)	117 (57.6)		
Central obesity (%)	121 (54.8)	58 (28.6)	29.73	<0.001[a]
SFA ⩾2.0 dm^2 (%)	120 (54.3)	74 (36.5)	13.58	<0.001[a]
VFA ⩾1.0 dm^2 (%)	158 (71.5)	63 (28.5)	94.96	<0.001[a]
Nation (%)				
Han	145 (65.6)	129 (63.5)		
Tibetan	72 (32.6)	70 (34.5)	0.20	0.91[a]
Other	4 (1.8)	4 (2.0)		
Smoking status (%)				
Never smoker	136 (61.5)	200 (98.5)		
Ex-smoker	14 (6.3)	0 (-)	88.07	<0.001[a]
Current smoker	71 (32.1)	3 (1.5)		
Drinking status (%)				
Never	82 (37.1)	177 (87.2)		
Occasionally	109 (49.3)	26 (12.8)	115.32	<0.001[a]
Regularly	30 (13.6)	0 (-)		
History of disease (%)				
Hypertension	25 (11.3)	7 (3.4)		
Diabetes	8 (3.6)	2 (1.0)	12.60	0.024[a]
Surgical history	20 (9.1)	34 (16.8)		
None	168 (76.0)	160 (78.8)		
BMI ($kg/m^{2)}$, mean (SD)	26.90(3.94)	24.12 (3.82)	54.24	<0.001[b]
WC (cm), mean (SD)	91.25 (9.60)	81.24 (10.02)	110.28	<0.001[b]
VFA (dm^2), mean (SD)	1.25 (0.44)	0.84 (0.39)	101.22	<0.001[b]
SFA (dm^2), mean (SD)	2.09(0.69)	1.85 (0.68)	13.12	<0.001[b]
SBP (mmHg), mean (SD)	129.33 (62.47)	118.73 (16.86)	5.48	0.020[b]
DBP (mmHg), mean (SD)	75.72 (10.89)	70.61(10.80)	23.50	<0.001[b]
Total protein (g/L), mean (SD)	73.79 (3.28)	73.10 (4.52)	3.27	0.071[b]
LYM (10^9/L), mean (SD)	2.17 (0.60)	1.99 (0.57)	10.14	0.002[b]
TG (mmol/L), mean (SD)	1.97 (1.60)	1.34 (1.53)	16.73	<0.001[b]
TC (mmol/L), mean (SD)	4.97 (0.94)	4.54 (0.96)	21.08	<0.001[b]
HDL-C (mmol/L), mean (SD)	1.16 (0.28)	1.37(0.30)	55.23	<0.001[b]
LDL-C (mmol/L), mean (SD)	3.14(0.83)	2.71 (0.82)	27.83	<0.001[b]
FPG (mmol/L), mean (SD)	4.91 (0.69)	4.78 (0.57)	4.75	0.030[b]

VFA, visceral fat area, SFA, subcutaneous fat area, SBP, systolic blood pressure, DBP, diastolic blood pressure, LYM, number of lymphocyte, FPG, fasting plasma glucose.

[a]*: P value of chi-square test,* [b]*: P value of ANOVA test*
