# Supplementary material for: Accumulated subcutaneous fat in abdomen is associated with long COVID-19 symptoms among non-hospitalized patients: a prospective observational study
Source: Front Med (Lausanne). 2024 Oct 14;11:1410559. doi: 10.3389/fmed.2024.1410559 (PMC11514070; doi:10.3389/fmed.2024.1410559)
Supplement: Supplementary file 2 [file Data_Sheet_2.pdf]

Supplemented Table 1-2. Symptoms of long-COVID-19 syndrome

Self-reported symptoms		Cases reported in follow-up period		
Systems	Symptoms	4th week	8th week	12th week
Respiratory symptoms	Cough	8	7	6
	Dyspnoea	5	3	2
Cardiovascular symptoms	Chest pains	7	4	4
	Palpitations	7	6	6
Neurological symptoms	Headache or dizziness	7	4	4
	Tinnitus	2	2	2
	Sleep disorders	19	17	16
	Muscle aches or pains	8	7	7
	Mental fog	2	2	2
Systemic symptoms	Fatigue	48	41	38
	Depression or anxiety	28	24	20
