# Supplementary material for: Accumulated subcutaneous fat in abdomen is associated with long COVID-19 symptoms among non-hospitalized patients: a prospective observational study
Source: Front Med (Lausanne). 2024 Oct 14;11:1410559. doi: 10.3389/fmed.2024.1410559 (PMC11514070; doi:10.3389/fmed.2024.1410559)
Supplement: Supplementary file 3 [file Data_Sheet_3.pdf]

Supplemented Table 2-1 Indicators of the population with long COVID-19 symptoms in different genders.

Gender	Characteristics	With symptoms	Without symptoms	*P* value
Male (n =214)	Case (%)	34 (15.9)	180 (84.1)	-
	With disease history (%)	15 (44.1)	38 (21.1)	0.008[a]
	Han (%)	30 (88.2)	108 (60.0)	<0.001[a]
	Tibetan (%)	2 (5.9)	70 (38.9)	
	Current smoker (%)	12 (35.3)	58 (32.2)	1.00[a]
	Regularly drinking (%)	26 (76.5)	158 (87.8)	0.75[a]
	Age (years), mean (SD)	38.24 (11.43)	41.47 (10.20)	0.098[b]
	BMI ($kg/m^{2)}$, mean (SD)	25.99 (3.68)	27.09 (4.03)	0.14[b]
	Obesity (%)	4 (11.8)	59 (32.8)	0.039[a]
	Overweight (%)	22 (64.7)	82 (45.6)	
	WC (cm), mean (SD)	97.44 (9.06)	91.78 (10.06)	0.003[b]
	Central obesity (%)	11 (32.4)	105 (58.3)	0.008[a]
	VFA (dm^2), mean (SD)	1.31 (0.24)	1.27 (0.44)	0.58[b]
	VFA ⩾ 1.0 dm^2 (%)	22 (64.7)	129 (71.7)	0.42[a]
	SFA (dm^2), mean (SD)	2.42 (0.64)	2.01 (0.67)	0.001[b]
	SFA ⩾ 2.0 dm^2 (%)	25 (73.5)	88 (48.9)	0.009[a]
	SBP (mmHg), mean (SD)	124.00 (15.65)	125.77 (16.02)	0.56[b]
	DBP (mmHg), mean (SD)	76.76 (8.06)	75.97 (11.44)	0.40[b]
	FPG (mmol/L), mean (SD)	5.06 (0.71)	4.91 (0.68)	0.24[b]
	TG (mmol/L), mean (SD)	2.10 (1.01)	1.97 (1.71)	0.65[b]
	TC (mmol/L), mean (SD)	5.22 (0.67)	4.94 (0.98)	0.11[b]
	HDL-C (mmol/L), mean (SD)	1.14 (0.24)	1.16 (0.29)	0.68[b]
	LDL-C (mmol/L), mean (SD)	3.38 (0.61)	3.13 (0.85)	0.09[b]
	Total protein (g/L), mean (SD)	74.48 (3.03)	73.57 (3.30)	0.15[b]
	LYM (10^9/L), mean (SD)	2.08 (0.59)	2.13 (0.57)	0.64[b]
Female (n = 194)	Case (%)	38 (19.6)	156 (80.4)	-
	With disease history (%)	10 (26.3)	33 (21.2)	0.52[a]
	Han (%)	26 (68.4)	94 (60.3)	0.46[a]
	Tibetan (%)	12 (31.6)	58 (37.2)	
	Current smoker (%)	0	3 (1.9)	1.00[a]
	Regularly drinking (%)	38 (100)	156 (100)	1.00[a]
	Age (years), mean (SD)	43.32 (12.90)	42.14 (10.79)	0.56[b]

BMI (kg/m^{2}), mean (SD)	24.97 (4.88)	23.93 (3.61)	0.14[b]
Obesity (%)	7 (18.4)	11 (7.1)	0.06[a]
Overweight (%)	17 (44.7)	46 (29.5)	
WC (cm), mean (SD)	84.63 (12.19)	80.52 (9.54)	0.026[b]
Central obesity (%)	16 (42.1)	39 (25.0)	0.045[a]
VFA (dm^2), mean (SD)	0.95 (0.35)	0.84 (0.51)	0.23[b]
VFA ⩾ 1.0 dm^2 (%)	13 (34.2)	31 (19.9)	0.08[a]
SFA (dm^2), mean (SD)	2.22 (0.93)	1.75 (0.58)	<0.001[b]
SFA ⩾ 2.0 dm^2 (%)	21 (55.3)	49 (30.8)	0.008[a]
SBP (mmHg), mean (SD)	119.13 (15.86)	118.31 (17.34)	0.79[b]
DBP (mmHg), mean (SD)	71.42 (11.25)	70.21 (10.85)	0.54[b]
FPG (mmol/L), mean (SD)	4.74 (0.80)	4.79 (0.52)	0.62[b]
TG (mmol/L), mean (SD)	0.96 (0.24)	1.37 (1.62)	0.12[b]
TC (mmol/L), mean (SD)	4.31 (0.85)	4.56 (0.96)	0.13[b]
HDL-C (mmol/L), mean (SD)	1.43 (0.27)	1.37 (0.31)	0.27[b]
LDL-C (mmol/L), mean (SD)	2.56 (0.74)	2.75 (0.85)	0.22[b]
Total protein (g/L), mean (SD)	72.25 (5.18)	73.47 (3.79)	0.77[b]
LYM (10^9/L), mean (SD)	1.87 (0.50)	2.00 (0.56)	0.20[b]

VFA, visceral fat area, SFA, subcutaneous fat area, SBP, systolic blood pressure, DBP, diastolic blood pressure, LYM, number of lymphocyte, FPG, fasting plasma glucose.

[a]*: P value of chi-square test,* [b]*: P value of ANOVA test*
