# Supplementary material for: Accumulated subcutaneous fat in abdomen is associated with long COVID-19 symptoms among non-hospitalized patients: a prospective observational study
Source: Front Med (Lausanne). 2024 Oct 14;11:1410559. doi: 10.3389/fmed.2024.1410559 (PMC11514070; doi:10.3389/fmed.2024.1410559)
Supplement: Supplementary file 4 [file Data_Sheet_4.pdf]

Supplemented Table 2-2 Indicators of the population with long COVID-19 symptoms in different nationalities.

Nationality	Characteristics	With symptoms	Without symptoms	*P* value
Han (n =258)	Case (%)	56 (21.7)	202 (78.3)	-
	Male (%)	30 (53.6)	108 (53.5)	1.0[a]
	With disease history (%)	20 (35.7)	39 (19.3)	0.01[a]
	Current smoker (%)	12 (21.4)	35 (17.3)	0.20[a]
	Regularly drinking (%)	8 (14.3)	17 (8.4)	0.34[a]
	Age (years), mean (SD)	40.09 (12.41)	42.78 (10.89)	0.12[b]
	BMI (kg/m$^{2)}$, mean (SD)	25.32 (4.48)	24.55 (3.39)	0.16[b]
	Obesity (%)	8 (14.3)	27 (13.4)	0.03[a]
	Overweight (%)	31 (55.4)	76 (37.6)	
	WC (cm), mean (SD)	85.82 (10.63)	83.20 (9.40)	0.07[b]
	Central obesity (%)	19 (33.9)	59 (29.2)	0.51[a]
	VFA (dm^2), mean (SD)	1.02 (0.38)	1.04 (1.34)	0.89[b]
	VFA ⩾ 1.0 dm^2 (%)	28 (50.0)	79 (39.1)	0.17[a]
	SFA (dm^2), mean (SD)	2.19 (0.80)	1.68 (0.58)	<0.001[b]
	SFA ⩾ 2.0 dm^2 (%)	31(55.4)	58 (28.7)	<0.001[a]
	SBP (mmHg), mean (SD)	123.61 (16.74)	127.38 (65.71)	0.67[b]
	DBP (mmHg), mean (SD)	75.55 (11.85)	73.67 (10.69)	0.26[b]
	FPG (mmol/L), mean (SD)	4.94 (0.85)	4.88 (0.60)	0.54[b]
	TG (mmol/L), mean (SD)	1.58 (0.95)	1.84 (2.06)	0.36[b]
	TC (mmol/L), mean (SD)	4.87 (0.87)	4.84 (1.02)	0.84[b]
	HDL-C (mmol/L), mean (SD)	1.28 (0.30)	1.28 (0.33)	0.97[b]
	LDL-C (mmol/L), mean (SD)	3.06 (0.79)	2.96 (0.89)	0.44[b]
	Total protein(g/L), mean (SD)	74.34 (4.01)	73.90 (3.34)	0.41[b]
	LYM (10^9/L), mean (SD)	2.12 (0.63)	2.08 (0.55)	0.66[b]
Tibetan (n = 142)	Case (%)	14 (9.9)	128 (90.1)	-
	Male (%)	2 (14.3)	70 (54.7)	0.004[a]
	With disease history (%)	5 (35.7)	31 (24.2)	0.35[a]
	Current smoker (%)	0	26 (100)	0.07[a]
	Regularly drinking (%)	0	5 (100)	0.41[a]
	Age (years), mean (SD)	46.43 (10.80)	40.26 (9.81)	0.029[b]
	BMI (kg/m$^{2)}$, mean (SD)	26.60 (3.75)	27.23 (4.66)	0.62[b]
	Obesity (%)	3 (21.4)	41 (32.0)	0.42[a]
	Overweight (%)	8 (57.1)	50 (39.1)	
	WC (cm), mean (SD)	90.36 (0.10)	91.60 (12.15)	0.71[b]
	Central obesity (%)	8 (57.1)	80 (62.5)	0.78[a]
	VFA (dm^2), mean (SD)	0.97 (0.32)	1.22 (0.52)	0.08[b]
	VFA ⩾ 1.0 dm^2 (%)	7 (50.0)	78 (60.9)	0.57[a]
	SFA (dm^2), mean (SD)	2.81 (0.73)	2.20 (0.61)	0.001[b]
	SFA ⩾ 2.0 dm^2 (%)	13(92.9)	75(58.6)	0.009[a]
	SBP (mmHg), mean (SD)	114.79 (9.21)	120.77 (16.62)	0.19[b]
	DBP (mmHg), mean (SD)	69.43 (7.24)	71.79 (12.08)	0.48[b]

	FPG (mmol/L), mean (SD)	4.75 (0.35)	4.80 (0.63)	0.10[b]
	TG (mmol/L), mean (SD)	1.20 (0.76)	1.43 (0.76)	0.29[b]
	TC (mmol/L), mean (SD)	4.29 (0.92)	4.67 (0.92)	0.14[b]
	HDL-C (mmol/L), mean (SD)	1.33 (0.30)	1.23 (0.29)	0.23[b]
	LDL-C (mmol/L), mean (SD)	2.57 (0.77)	2.94 (0.82)	0.11[b]
	Total protein(g/L), mean (SD)	70.21 (3.92)	72.86 (3.72)	0.01[b]
	LYM (10^9/L), mean (SD)	1.99 (0.61)	2.04 (0.57)	0.73[b]
**Other(n=4)**	Case (%)	2 (25)	6 (75)	-

VFA, visceral fat area, SFA, subcutaneous fat area, SBP, systolic blood pressure, DBP, diastolic blood pressure, LYM, number of lymphocyte, FPG, fasting plasma glucose.

[a]*: P value of chi-square test,* [b]*: P value of ANOVA test*
